# Cross-cultural translation of the Lysholm knee score in Chinese and its validation in patients with anterior cruciate ligament injury

**DOI:** 10.1186/s12891-016-1283-5

**Published:** 2016-10-19

**Authors:** W. Wang, L. Liu, X. Chang, Z. Y. Jia, J. Z. Zhao, W. D. Xu

**Affiliations:** 1 Department of Orthopedics, Chengdu Military General Hospital, Chengdu city, People’s Republic of China; 2 Department of Hepatic Surgery, Eastern Hepatobiliary Surgery Hospital, Shanghai, People’s Republic of China; 3 Department of urology, Changhai Hospital, Shanghai, People’s Republic of China; 4 Department of Orthopedics, Changhai Hospital, No.168, Changhai Road, Shanghai, 200433 People’s Republic of China; 5 Department of Orthopedics, No.401 Hospital, Jinan Military Region of PLA, Qingdao city, Shandong province People’s Republic of China

**Keywords:** Lysholm knee score, Quality of Life, Validation, Adaptation, Psychometrics

## Abstract

**Background:**

The Lysholm Knee Score (LKS) is widely used and is one of the most effective questionnaires employed to assess knee injuries. Although LKS has been translated into multiple languages, there is no Chinese version even though China has the largest population of patients with knee-joint injuries. The objective of our study was to develop the Chinese version of LKS (C-LKS) and assess its reliability, validity and responsiveness in Chinese patients with anterior cruciate ligament (ACL) injuries.

**Methods:**

Study participants were mainly recruited among patients with ACL injuries scheduled for arthroscopic ACL reconstruction at our hospital. First, we developed the C-LKS in a five-step translation and cross-cultural adaptation procedure. Next, we calculated the Cronbach’s alpha, intraclass correlation coefficient (ICC), Pearson’s correlation coefficient (*r*), effect size (ES), and standardized response mean (SRM) to evaluate the reliability, validity, and responsiveness of C-LKS respectively.

**Results:**

Overall, 126 patients with ACL injuries successfully completed the questionnaires. Acceptable internal consistency (Cronbach’s alpha = 0.726) as well as excellent test-retest reliability (ICC = 0.935) was found for C-LKS. Good or moderate correlation (*r* = 0.514–0.837) was determined among C-LKS and International Knee Documentation Committee Subjective Knee Form (IKDC), Western Ontario and McMaster Universities Osteoarthritis Index (WOMAC), physical subscales of SF-36; C-LKS also had fair or moderate correlation (*r* = 0.207–0.462) with the other subscales of SF-36, which adequately illustrated that good validity was included in C-LKS. In addition, good responsiveness was also observed in C-LKS (ES = 1.36,SRM = 1.26).

**Conclusions:**

We have shown that our developed C-LKS questionnaire is reliable, valid and responsible for the evaluation of Chinese-speaking patients with ACL injuries and it would be an effective instrument.

## Background

The knee is the biggest and most complex joint in the human body and is highly prone to injury [1]. With outdoor physical activities being performed more frequent and more complex in modern times, the incidence of knee injuries rises as well, particularly in young people and athletes [2].

With an increasing concern on treatment of knee injury and rehabilitation, a number of questionnaires have been put into practice that may help doctors evaluate the severity of knee injury and recovery after treatment, such as the Oxford Knee Score (OKS) [3], International Knee Documentation Committee Subjective Knee Form (IKDC) [4], Tegner Activity Scale [5], Western Ontario and McMaster Universities Osteoarthritis Index (WOMAC) [6], Knee Injury and Osteoarthritis Outcome Score (KOOS) [7], and the Lysholm knee score [8]. These questionnaires tend to focus on clinical manifestations and the patient’s subjective feelings to evaluate the impact of injury on knee function and overall quality of life, which may help to offer better diagnosis and treatment options.

The Lysholm knee score, published in 1982 [8], was initially used to evaluate the functional state of the patient after anterior cruciate ligament (ACL) injury, and follow-up researches have proven its value in functional evaluation for other knee injuries, including patellofemoral pain syndrome [9], meniscal injuries [10], medical patellar plica syndrome [11], patellar dislocation [12], and various chondral disorders [13]. Compared with other knee scoring scales, the Lysholm knee score has many advantages. For example, OKS is only applicable for functional evaluation in knee osteoarthritis, and IKDC and Tegner Activity Scale are only used to evaluate knee ligament [3–6]. WOMAC and KOOS have 24 and 42 items, respectively, and the average time to complete the questionnaires ranges from 5 to 10 min [14], which is considered lengthy in the realm of knee questionnaires. The Lysholm knee score, on the other hand, has broad applicability and has only eight items that can be completed by patients in a short period of time [8, 15].

Because of these benefits, the Lysholm knee score has been used by clinicians and researchers for over three decades. During the past 5 years, over 700 articles cited in PubMed have reported outcomes using the Lysholm knee score. Additionally, the original English version of Lysholm knee score has been further translated and validated into many languages [1, 15, 16]. Despite China being the most populous country and Mandarin being the most prevalent language in the world, but a Chinese version is still absent. Thus, it is essential for presumably the largest patient population with knee injuries in the world to have.

When a reliable, valid questionnaire is being used in populations with different cultures, it is necessary to test the psychometric properties of the questionnaire, rather than to simply translate the content, in order to avoid evaluation errors caused by cultural differences [17, 18]. Hence, we aimed to translate and adapt the Lysholm knee score into a Chinese version (C-LKS) and to evaluate the psychometric properties of the C-LKS in a cohort of native Chinese-speaking patients with ACL injuries. These psychometric properties assessed were acceptability, reliability, validity and responsiveness.

## Methods

### Translation and cross-cultural adaptation

Translation of the English original Lysholm knee score followed previously published guidelines [19, 20]. The entire process consisted of five steps: 1) Forward translation from English to Chinese by two bilingual translators independently, who are native Chinese speakers and well conversant in English. One of the translators is an orthopedic surgeon in our department (the author, WW), the other is a full-time translator (RL) with no medical background, and is not informed of our investigation; 2) Revision and modification of the questionnaire regarding language expressions and cultural differences was discussed by the two forward translators and other research members. A primary version of C-LKS was then obtained; 3) Backward translation by two independent native English translators (FA and GD) who are well conversant in Chinese. The primary version of C-LKS was translated from Chinese to English. The two translators had medical backgrounds, with no knowledge of the original Lysholm knee score; 4) All researchers and translators convened and had discussions to solve any discrepancies, ambiguities and other language expression issues that existed in the questionnaire, and the pre-final version of C-LKS was obtained; and 5) Twenty patients with ACL injuries patients were invited to complete the pre-final C-LKS for assessment, and feedbacks were collected. The researchers met once more to make final adjustments according to these feedbacks and the final version of C-LKS was obtained.

### Patients and data collection

Owing to the fact that the Lysholm knee score was initially designed for patients with ACL injuries [8, 15, 21], we also recruited patients with ACL injuries to minimize deviations. Cases involved in the present study were mainly recruited among patients with ACL injuries scheduled for arthroscopic ACL reconstruction at our hospital [22]. Our inclusion criteria were: Aged over 16 years old with independent signing authority; Chinese as the first language with adequate capability to read and complete a questionnaire; and a definitive diagnosis of ACL injury as determined by arthroscopy [21]. Patients with other complicated knee injuries, such as meniscal injuries or patellar dislocation; with a history of lower limb or spine surgery; patients who had surgery within a month of the study; and patients with a history of systemic disease and/or malignancy, were excluded from this study. Our study met the quality criteria proposed by Terwee and associates [23] for measurement properties of health status questionnaires, which required the results from at least 100 patients to perform internal consistency analysis and from at least 50 patients for floor or ceiling effects, reliability, and validity analyses. All patients involved in the study had thoroughly read and signed the informed consent. This study was approved by the ethics committee in the local hospital (No. CHEC2013-199).

The patients provided demographic information, such as sex, age and weight, on the first day of enrollment and independently completed the four questionnaires, C-LKS, WOMAC, IKDC, and Medical Outcomes Study Short-Form 36 (SF-36) in a quiet meeting room, followed by a C-LKS again 1 week later before receiving reconstruction surgery, in order to evaluate the test-retest reliability of the questionnaire. Patients were also reached via mail or telephone 6 months postoperatively to complete the C-LKS a time to help evaluate the responsiveness of the questionnaire.

### Questionnaires

The Lysholm knee score had eight items that evaluated walking gait, frequency of knee locking, frequency of pain, stair climbing, need for external support, body stability, joint swelling, and squatting ability. A total score (ranged 0 to 100 points) was calculated from the patient’s answers that best reflected his/her functional state. A lower score was indicative of poor knee function [8].

IKDC, first published in 2001, consists of 10 dimensions and evaluates the clinical symptoms, activity states, and function of the patient. The answers are checked accordingly, and the final score (range 0 to 100 points) is then calculated by a certain formula. WOMAC is a self-reported questionnaire specifically designed to evaluate the functional state of the knee or hip. It consists of 24 items divided into three subscales, namely, Pain (5 items), Stiffness (2 items), and Physical functioning (17 items). The final score (range 0 to 96 points) is the sum of all items. Unlike the majority of other questionnaires, a lower score represents a better functional state of the joint. SF-36 is a generic questionnaire for quality of life comprised of 35 items and eight dimensions that evaluate mental health and physiological and social functioning. Each dimension has its unique scoring system, and the final score is converted to percentages. A lower SF-36 score suggests a poorer quality of life or functional state. The above three scales have existed Chinese versions and are proven with excellent reliability, validity and responsiveness [24–26].

### Psychometric assessments and statistical analysis

To evaluate the acceptability of the questionnaire in the general population, we asked each patient in our study cohort if they had any difficulties understanding the content. We also calculated the miss rate of every item, and a >5 % miss rate of a certain item suggested an existing problem regarding acceptability or comprehension [27]. We also recorded the average time required to complete the questionnaire.

A distribution of scores was analyzed to determine whether a ceiling or floor effect existed. A result of <30 % of either results was considered acceptable [21].

Reliability tests included evaluations for test-retest reliability and internal consistency. Test-retest reliability was performed by comparing the former two C-LKS results of which the evaluation norm was the intraclass correlation coefficient (ICC), which was derived from a two-way analysis of variance (ANOVA) in a random effects model. These results displayed good reliability when ICC > 0.6, and excellent reliability when ICC was >0.8 [28]. Cronbach’s alpha was used to evaluate internal consistency when it was >0.7, >0.8 and >0.9, the questionnaire was regarded as having acceptable, good and excellent internal consistency, respectively [23]. We further depicted Bland-Altman plots to observe for systematic error between the investigations [29, 30].

Validity tests were performed particularly evaluating content validity, construct validity and external validity. For content validity, a rehabilitation expert (QW) and three orthopedic experts (ZW, YK and the author WX) helped to assess the understanding and relevance of all of the items in the C-LKS. Good construct validity referred to a questionnaire that correlated well with measures of the same construct (convergent validity), while poorly correlated with measures of different constructs (divergent validity) [31]. Therefore, we initially assumed that the score of C-LKS should correlate well with the physical subscales (i.e. Physical Functioning, Role-Physical, and Bodily Pain) of SF-36, but correlate poorly with other subscales (Vitality, Role-Emotional, Mental Health, Social Function and General Health) of SF-36. Based upon such assumption, the Pearson’s correlation coefficient (*r*) of C-LKS with subscales of the SF-36, WOMAC and IKDC was calculated. The construct validity of C-LKS was evaluated by comparing the compatibility of the results with our initial assumption. The correlations were judged either as poor (*r =* 0–0.2), fair (*r* = 0.2–0.4), moderate (*r* = 0.4–0.6), very good (*r =* 0.6–0.8), or excellent (*r =* 0.8–1.0) [31]. We also calculated the Cohen’s Kappa (*k*) coefficient between the results of C-LKS and IKDC to evaluate external validity, and *k* > 0.60 was thought to be necessary for an acceptable external validity [32].

Finally, we evaluated the responsiveness of C-LKS by comparing questionnaire results before treatment and 6 months after treatment. Effect size (ES) and standardized response mean (SRM) were the two indices used to evaluate responsiveness. SRM was defined as the mean change between these time points divided by the SD of this change. ES was defined as the mean change between preoperative results and 6 month postoperative results divided by the SD of the preoperative C-LKS score [33]. A greater value of ES and SRM suggested a better responsiveness of C-LKS.

Statistical Package for the Social Sciences, version 20.0 (SPSS, Chicago, IL, USA) was used for statistical analysis. Data are presented as mean ± standard deviation (SD). ICC values are reported with 95 % confidence intervals (CIs). *P* value of 0.05 or less was considered statistically significant.

## Results

### Participants

A total of 159 patients with ACL injuries (89 males and 70 females) admitted to our hospital from January 2013 to May 2014 were invited to participate in our study. A total of 126 (79.2 %, 69 males and 57 females) of those invited patients agreed to participate in our study. They all had completed the C-LKS three times in the following 6 months with no withdrawn cases. Detailed demographic information was listed in Table 1.

**Table 1:** Characteristics of participants

Characteristics	Total sample (*N* = 126)	Male (*n*_1_ = 69)	Female (*n*_2_ = 57)	*P* value^a^
Age(years; mean ± SD)	25.9 ± 7.6	25.4 ± 6.9	26.5 ± 8.4	0.419
Range	16–58	16–47	17–58	
Age groups; number(%)				0.755
≦20	25 (19.8 %)	15 (21.7 %)	10 (17.5 %)	
20–30	67 (54.0 %)	37 (55.1 %)	30 (52.6 %)	
30–40	21 (16.7 %)	11 (15.9 %)	10 (17.5 %)	
≧40	13 (9.5 %)	6 (7.2 %)	7 (12.3 %)	
Affected side; number(%)				0.759
Right	66 (52.4 %)	37 (53.6 %)	29 (50.9 %)	
Left	60 (47.6 %)	32 (46.4 %)	28 (49.1 %)	
BMI (Kg/m^2^; mean ± SD)	24.3 ± 3.5	23.9 ± 3.2	24.8 ± 3.8	0.141
ACL injury duration (months; mean ± SD)	5.5 ± 3.4	5.8 ± 3.2	5.1 ± 3.7	0.272
Range	0.5–12	1–12	0.5–11	

*ACL* anterior cruciate ligament, *BMI* body mass index

^a^Calculated by Student’s t-tests for continuous variables and Chi^2^ tests for categorical variables between males and females

### Translation and cross-culture adaptation process

Forward and backward translations went smoothly. The most important modification in the prefinal C-LKS compared with the original English version was that the corresponding points marked beside the items and answers were removed, and other detail issues were also resolved. During the pre-evaluation period, more than half of the patients claimed to have difficulty understanding the terminology in the questionnaire, such as “locking” and “instability”; therefore, explanations with simple language were attached beside the questions in the final version of C-LKS.

### Acceptability and score distribution

In our formal investigation, no respondents claimed difficulties understanding the questionnaire after completing C-LKS for the first time, and the answer rates for all questions were 100 % with no missed questions. The average time to complete the questionnaire was 79 ± 21 s.

Overall, C-LKS had no ceiling effect (1.6 %) or floor effect (0.8 %), but the ceiling effect did exist for items three (“*Locking*”) and six (“*Support*”) (Table 2).

**Table 2:** Score distribution and floor-ceiling effects of the C-LKS

Item	Mean ± SD	Observed range	Theoretical range	Floor effect (%)^a^	Ceiling effect (%)^a^
Overall scale	58.14 ± 15.25	0–100	0–100	0.8	1.6
Limp	3.27 ± 1.38	0–5	0–5	9.5	28.6
Locking	11.30 ± 4.27	0–15	0–15	3.2	50.8
Pain	13.13 ± 4.68	0–25	0–25	4.0	4.8
Stair climbing	4.89 ± 3.46	0–10	0–10	11.1	23.0
Support	3.98 ± 1.59	0–5	0–5	4.8	69.0
Instability	13.53 ± 4.15	0–25	0–25	2.4	3.2
Swelling	4.62 ± 3.05	0–10	0–10	8.7	14.3
Squatting	3.52 ± 1.32	0–5	0–5	7.9	1.6

^a^Percentage of patients with the worst (floor effect) and the best (ceiling effect) condition

### Reliability

**Fig. 1:**
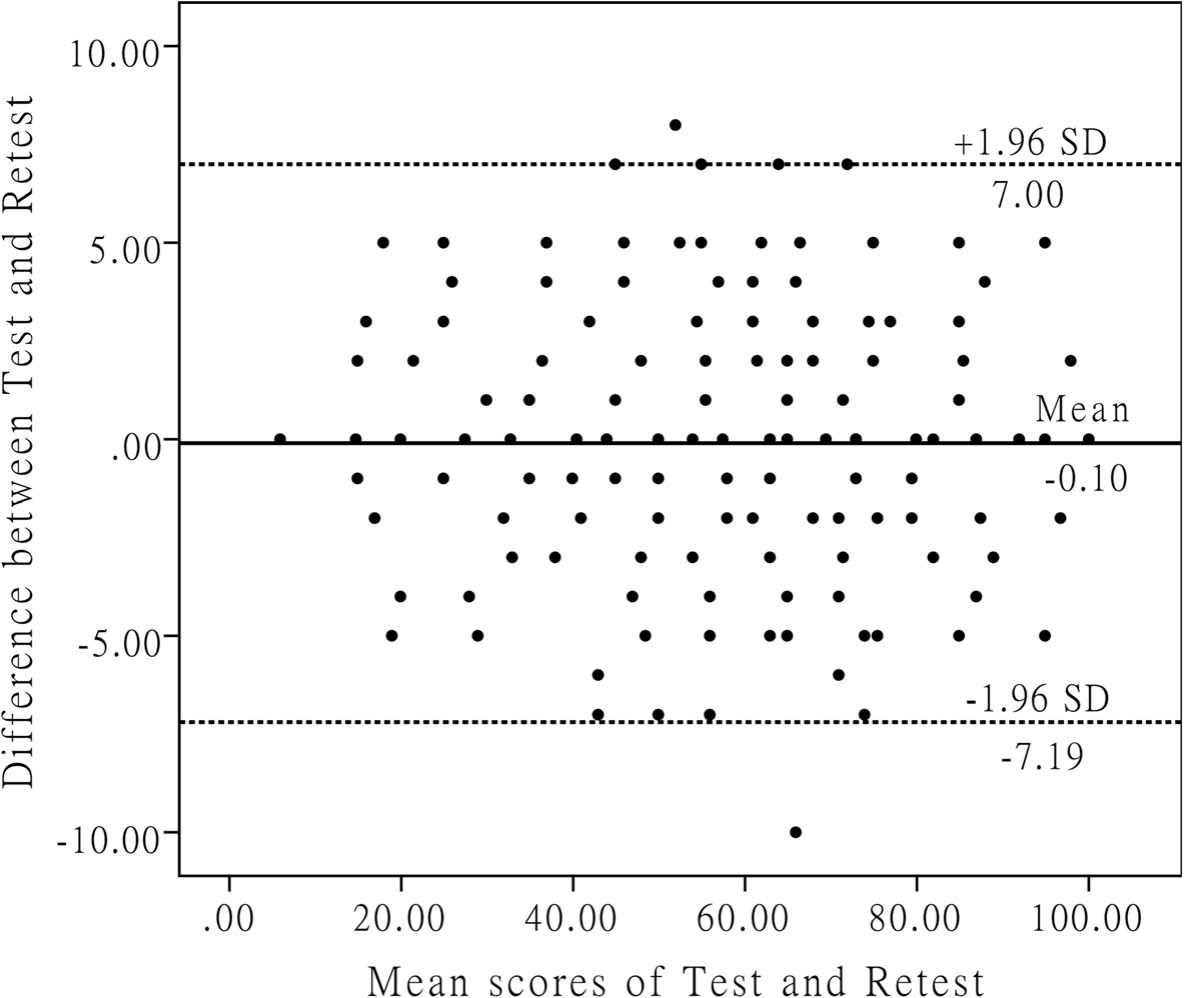
These are Bland-Altman plots of test-retest reliability of the C-LKS. Each data point indicates how the difference between the two test sessions for an individual patient compares to the mean of the two sessions for scores of each C-LKS. The interval of two sessions was 2 weeks. The dashed line shows the 95 % (±1.96 SD) limits of agreement

The Cronbach’s alpha of C-LKS was 0.726, indicative of acceptable internal consistency. The overall test-retest reliability was “excellent” (ICC = 0.935), and the test-retest reliability for each item ranged from acceptable, good to excellent (ICC = 0.770–0.994) (Table 3). The Bland-Altman plots revealed no systematic error in the first two questionnaires (Fig. 1), which also confirmed and highlighted good test-retest agreement of C-LKS.

**Table 3:** Test-retest reliability and responsiveness of the C-LKS^a^

Item	1st-Test (mean ± SD)^b^	2nd-Test (mean ± SD)^b^	3rd-Test (mean ± SD)^b^	ICC (CI range)	ES	SRM
Overall scale	58.14 ± 15.25	57.98 ± 13.94	78.98 ± 10.36	0.935 (0.909–0.954)	1.36	1.26
Limp	3.27 ± 1.38	3.11 ± 1.38	4.43 ± 0.91	0.855 (0.797–0.896)	0.84	0.76
Locking	11.30 ± 4.27	11.04 ± 4.66	13.17 ± 3.93	0.770 (0.689–0.833)	0.44	0.32
Pain	13.13 ± 4.68	13.57 ± 4.33	17.62 ± 4.85	0.878 (0.830–0.913)	0.96	0.91
Stair climbing	4.89 ± 3.46	4.75 ± 3.82	9.10 ± 1.89	0.757 (0.672–0.804)	1.21	1.11
Support	3.98 ± 1.59	3.99 ± 1.56	4.95 ± 0.38	0.994 (0.991–0.995)	0.62	0.59
Instability	13.53 ± 4.15	13.45 ± 3.71	17.38 ± 4.23	0.936 (0.910–0.955)	0.93	0.89
Swelling	4.62 ± 3.05	4.70 ± 3.20	7.40 ± 2.90	0.813 (0.744–0.865)	0.91	0.84
Squatting	3.52 ± 1.32	3.37 ± 1.42	4.93 ± 0.26	0.768 (0.686–0.831)	1.07	1.04

*ICC* intra-class correlation coefficient, *ES* effect size; SRM: standardized response mean, *CI* 95 % confidence interval

^a^The sample size for the analysis of test-retest reliability and responsiveness was 126

^b^The 1st-Test was conducted at the beginning of this research, the 2nd-Test was conducted 1 week later to calculate the test-retest reliability (ICC) of the C-LKS, and the 3rd-Test was conducted 6 months later to calculate the responsiveness (ES, SRM) of the C-LKS

### Validity

With the analysis and evaluation of content by rehabilitation and orthopedic experts, the questionnaire was regarded to have good content validity, and the information acquired from the questions was adequate to evaluate the functional state of patients with ACL injuries. Therefore, no addition or deletion of items was recommended.

Relevant data for construct validity evaluation are listed in Table 4, and the data were highly consistent with our presumed results. The correlation between C-LKS and IKDC was excellent (*r =* 0.734–0.811), while that with the three subscales of WOMAC was very good or excellent (*r =* 0.634–0.811), and that with the physical subscales of SF-36 were moderate or very good (*r =* 0.514–0.709), but that with other subscales of SF-36 was fair or moderate (*r =* 0.207–0.462) or no significant correlations (*P* > 0.05). These results suggested that C-LKS had good construct validity.

**Table 4:** Construct validity of the C-LKS^a^

Scales	Correlation coefficient (*r*)^b^	*P* value
IKDC	0.837	<0.0001
WOMAC		
Pain	- 0.773	<0.0001
Stiffness	- 0.634	<0.0001
Physical Function	- 0.811	<0.0001
SF-36		
Physical Function	0.709	<0.0001
Role-Physical	0.514	<0.0001
Bodily Pain	0.676	<0.0001
General Health	0.462	<0.0001
Vitality	0.303	0.001
Social Function	0.366	<0.0001
Role-Emotional	0.207	0.020
Mental Health	0.163	0.068

*IKDC* International Knee Documentation Committee Subjective Knee Form, *SF-36* Short-Form 36, *WOMAC* Western Ontario and McMaster Universities Osteoarthritis Index

^a^The sample size for the analysis of construct validity was 144

^b^Calculated by the Pearson correlation of the Simplified Chinese version of C-LKS with IKDC, WOMAC and SF-36

Lastly, the Cohen’s Kappa (*k*) coefficient between the results of C-LKS and IKDC was 7.3, which suggested that C-LKS had acceptable external validity.

### Responsiveness

Finally, we evaluated the responsiveness of C-LKS by comparing the questionnaires completed before and after ACL reconstruction. Relevant data are listed in Table 3. In general, the average score increased by 21 points after treatment, the ES (1.36) and SRM (1.26) values both exceeded 1.00, suggesting good responsiveness to the questionnaire.

## Discussion

Functional or quality of life questionnaires are critical tools in clinical investigations. Researchers are able to compare data with other questionnaires and evaluate the functional state of patients. China has witnessed a rapid development of clinical scientific research over time, and a large amount of relevant articles are being published each year, which not only owes to the largest patient population of China, but also to the great attention and support from the government [31]. Therefore, effective questionnaires are very much in need to better support these massive clinical researches. The Lysholm knee score is one of the most widely applied questionnaires in evaluating the functional state after knee injuries with excellent reliability, validity and responsiveness [9, 10, 12, 13, 15, 21, 34–36]. Therefore, we believe that the translation of such scale for the country with the largest patient population is of great significance, which is also the major objective of our study.

During the course of C-LKS development, we removed the corresponding scores noted beside the questions and answers, because we believed that they may affect patient answers if the patients could see the point values. To note, removal of such markings did not influence their understanding of the content. Meanwhile, we also consulted the suggestions from Derya et al [15] to convert the units of walking distance in item 3 (“*Pain*”) from km to min in order to better estimate patient’s walking capability. Additionally, because China is a developing country, the average education level is still relatively low, and thus some terminologies in the questionnaire, such as “*locking*” and “*instability*” may confuse the patients, as reflected by many of them in the pretest phase. Hence, detailed explanations were added beside these items. “*Locking*” was explained as “A loss of activity with a ‘locked’ sense of the knee when walking or squatting, usually with marked pain”. No further difficulties in understanding the words or content were reflected in the follow-up research.

C-LKS had an acceptable internal consistency (Cronbach’s alpha = 0.726), which was consistent with other studies (Cronbach’s alpha = 0.650–0.729) [10, 12, 13, 15, 21]. It also had excellent test-retest reliability (ICC = 0.935), also consistent with other studies (ICC = 0.820–0.950) [10, 12, 13, 15, 21, 34]. Notably, the ICC value associated with item 5 was very close to 1 (0.994), possibly because of the fact that the usage of crane within a week may not differ at all. Furthermore, we believe that the 1-week margin for test-retest reliability was subtle because the functional state would not markedly change within a week. Additionally, this did not exceed the time interval adopted in other studies (4 to 24 days) [13], and moreover, this timeframe equaled the time required to wait for reconstruction with no additional treatment, thus reducing related errors.

The correlation of C-LKS with the WOMAC, IKDC and SF-36 is consistent with our presumptions, showing good construct validity. This result corroborated previous studies [12, 13, 15, 21, 37]. The correlation between C-LKS and IKDC was the strongest (*r =* 0.836), which may be because of the fact that IKDC was also designed for patients with ACL injuries, and the cases included in this study were also ACL injury patients. Despite that the physical subscales in SF-36 are to evaluate the functional states of activity, they do not have strong correlation with C-LKS (*r* = 0.514–0.709). One possible reason is that as a generic scale, SF-36 has a markedly lower degree of accuracy when evaluating the functional state for certain patients, compared with other specific scales [38]. Particularly, the mental subscales of the SF-36 correlated weakly or not at all with C-LKS (*r* = 0.207–0.303 or *P* > 0.05). Such a finding may be expected because the mental state of a patient is affected by many factors in life.

The responsiveness to a questionnaire is an important determining factor for prospective clinical investigations. Our results showed good responsiveness of C-LKS (ES = 1.36, SRM = 1.26), suggesting that it could sensitively detect the change in functional state after ACL reconstruction surgery. The ES and SRM values are slightly greater than previous studies (ES = 0.87–1.20, SRM = 0.77–1.10) [10, 13, 21, 34]. This may be because of the fact that all of our study participants underwent arthroscopic ligament reconstruction, while conservative treatment was included in other studies, which may have affected the degree of difference in the improvement of functional state. Furthermore, only the ES and SRM values of item 2 (“*Locking*”) were below 0.5, which may not be issues from our translation or modification, because other studies showed similar results (ES = 0.28–0.55, SRM = 0.23–0.50) [10, 13, 21]. Thus, we posit that surgery or any other conservative treatment is not likely to significantly improve locking symptom.

However, some limitations of the present study should be noted. First, a relatively small sample size may not perfectly represent the entire Chinese knee injury patient population, but the information from 126 patients is adequate to evaluate psychometric properties [23], and is no less than that of similar studies [1, 15, 16]. Therefore, the reliability of our study would not be affected by the sample volume; second, the language we chose to adapt into is Chinese which does not cover the whole population because China is a multi-group nation and each minority group speaks their own tongue, which should be noted when applying the questionnaire.

## Conclusions

In summary, we have successfully translated and modified the Lysholm knee scale into Chinese version, and proved good reliability, validity and responsiveness. Therefore, we suggest the application of the translated C-LKS for Chinese-speaking patients to evaluate the functional state after ACL injury to better collect data required for doctors or researchers.

## Abbreviations

ACL: Anterior Cruciate Ligament
C-LSK: Chinese version of Lysholm knee score
IKDC: International Knee Documentation Committee Subjective Knee Form
KOOS: Knee Injury and Osteoarthritis Outcome Score
OKS: Oxford Knee Score
WOMAC: Western Ontario and McMaster Universities Osteoarthritis Index

## References

[1] Peccin MS, Ciconelli R, Cohen M (2006). Specific questionnaire forknee symptoms: The “Lysholm Knee Scoring Scale”. Translationand validation into Portuguese. Acta Orthop Bras.

[2] Ingram JG, Fields SK, Yard EE, Comstock RD (2008). Epidemiology ofknee injuries among boys and girls in US high school athletics. Am J Sports Med.

[3] Dawson J, Fitzpatrick R, Murray D, Carr A (1998). Questionnaire on the perceptions of patients about total knee replacement. J Bone Joint Surg (Br).

[4] Irrgang JJ, Anderson AF, Boland AL, Harner CD, Kurosaka M, Neyret P, Richmond JC, Shelbone KD (2001). Development and validation of the international knee documentation committee subjective knee form. Am J Sports Med.

[5] Tegner Y, Lysholm J (1985). Rating systems in the evaluation of kneeligament injuries. Clin Orthop Relat Res.

[6] Bellamy N, Buchanan WW, Goldsmith CH, Campbell J, Stitt LW (1988). Validation study of WOMAC: a health status instrumentfor measuring clinically important patient relevant outcomes toantirheumatic drug therapy in patients with osteoarthritis of thehip or knee. J Rheumatol.

[7] Roos EM, Roos HP, Lohmander LS, Ekdahl C, Beynnon BD (1998). Knee Injury and Osteoarthritis Outcome Score (KOOS): development of a self-administered outcome measure. J Orthop Sports Phys Ther.

[8] Lysholm J, Gillquist J (1982). Evaluation of knee ligament surgeryresults with special emphasis on use of a scoring scale. Am J Sports Med.

[9] Bengtsson J, Mollborg J, Werner S (1996). A study for testing the sensitivity and reliability of the Lysholm knee scoring scale. Knee Surg Sports Traumatol Arthrosc.

[10] Briggs KK, Kocher MS, Rodkey WG, Steadman JR (2006). Reliability, validity, and responsiveness of the Lysholm knee score and Tegner activity scale for patients with meniscal injury of the knee. J Bone Joint Surg Am.

[11] Denti M, Monteleone M, Berardi A, Arosio A (1994). Medial patellar synovial plica syndrome: the influence of associated pathology on long-term results. Chir Organi Mov.

[12] Paxton EW, Fithian DC, Stone ML, Silva P (2003). The reliability and validity of knee-specific and general instruments in assessing acute patellar dislocation outcomes. Am J Sports Med.

[13] Kocher MS, Steadman JR, Briggs KK, Sterett WI, Hawkins RJ (2004). Reliability, validity, and responsiveness of the Lysholm knee scale for various chondraldisorders of the knee. J Bone Joint Surg Am.

[14] Collins NJ, Misra D, Felson DT, Crossley KM, RoosEM. Measures of knee function: International Knee DocumentationCommittee (IKDC) Subjective Knee Evaluation Form, KneeInjury and Osteoarthritis Outcome Score (KOOS), Knee Injuryand Osteoarthritis Outcome Score Physical Function Short Form(KOOS-PS), Knee Outcome Survey Activities of Daily LivingScale (KOS-ADL), Lysholm Knee Scoring Scale, Oxford KneeScore (OKS), Western Ontario and McMaster UniversitiesOsteoarthritis Index (WOMAC), Activity Rating Scale (ARS),and Tegner Activity Score (TAS). Arthritis Care Res (Hoboken). 2011; 63(suppl 11): S208–228. 10.1002/acr.20632 PMC4336550 22588746

[15] Celik D, Coşkunsu D, Kiliçoğlu O (2013). Translation and cultural adaptation of the Turkish Lysholm knee scale: ease of use, validity, and reliability. Clin Orthop Relat Res.

[16] Wirth B, Liffert F, de Bruin ED (2011). Development and evaluation of a German version of the Lysholm score for measuring outcome after anterior cruciate ligamentinjuries in German. Sportverletz Sportschaden.

[17] Pynsent PB (2001). Choosing an outcome measure. J Bone Joint Surg (Br).

[18] Zheng W, Li J, Zhao J, Liu D, Xu W (2014). Development of a Valid Simplified Chinese Version of the Oxford Hip Score in Patients With Hip Osteoarthritis. Clin Orthop Relat Res.

[19] Guillemin F, Bombardier C, Beaton D (1993). Cross-cultural adaptation of health-related quality of life measures: literature review and proposed guidelines. J Clin Epidemiol.

[20] Beaton DE, Bombardier C, Guillemin F, Ferraz MB (2000). Guidelines for the process of cross-cultural adaptation of self report measures. Spine.

[21] Briggs KK, Lysholm J, Tegner Y, Rodkey WG, Kocher MS, Steadman JR (2009). The reliability, validity, and responsiveness of the Lysholm score and Tegner activity scale for anterior cruciate ligament injuries of the knee: 25 years later. Am J Sports Med.

[22] Ahn JH, Lee SH (2007). Anterior cruciate ligament double-bundle reconstruction with hamstring tendon auto grafts. Arthroscopy.

[23] Terwee CB, Bot SD, de Boer MR, van der Windt DA, Knol DL, Dekker J, Bouter LM, de Vet HC (2007). Quality criteria were proposed for measurement properties of health status questionnaires. J Clin Epidemiol.

[24] Fu SN, Chan YH (2011). Translation and validation of Chinese version of International Knee Documentation Committee Subjective Knee Form. Disabil Rehabil.

[25] Xie F, Li SC, Gorerr R, Tarride JE, O’Reilly D, Lo NN, Yeo SJ, Yang KY, Thumboo J (2008). Validation of Chinese Western Ontario and McMaster Universities Osteoarthritis Index (WOMAC) in patients scheduled for total knee replacement. Qual Life Res.

[26] Li L, Wang HM, Shen Y (2003). Chinese SF-36 Health Survey: translation, cultural adaptation, validation, and normalisation. J Epidemiol Community Health.

[27] Coste J, Fermanian J, Venot A (1995). Methodological and statistical problems in the construction of composite measurement scales: a survey of six medical and epidemiological journals. Stat Med.

[28] Landis JR, Koch GG (1977). The measurement of observer agreement for categorical data. Biometrics.

[29] Bland JM, Altman DG (1986). Statistical methods for assessing agreement between two methods of clinical measurement. Lancet.

[30] Bland JM, Altman DG (1999). Measuring agreement in method comparison studies. Stat Methods Med Res.

[31] Wei X, Wang Z, Yang C, Wu B, Liu X, Yi H, Chen Z, Wang F, Bai Y, Li J (2012). Development of a simplified Chinese version of the hip disability and osteoarthritis outcome score (HOOS): cross-cultural adaptation and psychometric evaluation. Osteoarthr Cartil.

[32] Rolfes L, Kolfschoten J, van Hunsel F, van Puijenbroek E (2016). The validity and reliability of a signal impact assessment tool. Pharmacoepidemiol Drug Saf.

[33] Husted JA, Cook RJ, Farewell VT, Gladman D (2000). Methods for assessing responsiveness: a critical review and recommendations. J Clin Epidemiol.

[34] Marx RG, Jones EC, Allen AA, Altchek DW, O’Brien SJ, Rodeo SA, Williams RJ, Warren RF, Wickiewicz TL (2001). Reliability, validity, and responsiveness of four knee outcome scales for athletic patients. J Bone Joint Surg Am.

[35] Risberg MA, Holm I, Steen H, Beynnon BD (1999). Sensitivity to changes over time for the IKDC form, the Lysholm score, and the Cincinnati knee score. A prospective study of 120 ACL reconstructed patients with a 2-year follow-up. Knee Surg Sports Traumatol Arthrosc.

[36] Briggs KK, Steadman JR, Hay CJ, Hines SL (2009). Lysholm score and Tegner activity level in individuals with normal knees. Am J Sports Med.

[37] Kim JG, Ha JK, Lee JY, Seo SS, Choi CH, Lee MC (2013). Translation and validation of the korean version of the international knee documentationcommittee subjective knee form. Knee Surg Relat Res.

[38] Patrick DL, Deyo RA (1989). Generic and disease-specific measures in assessing health status and quality of life. Med Care.

